# Assessment of Disability Progression Independent of Relapse and Brain MRI Activity in Patients with Multiple Sclerosis in Poland

**DOI:** 10.3390/jcm10040868

**Published:** 2021-02-19

**Authors:** Katarzyna Kapica-Topczewska, François Collin, Joanna Tarasiuk, Agata Czarnowska, Monika Chorąży, Anna Mirończuk, Jan Kochanowicz, Alina Kułakowska

**Affiliations:** 1 Department of Neurology, Medical University of Bialystok, 15-276 Bialystok, Poland; amirtarasiuk@wp.pl (J.T.); agtczarnowska@gmail.com (A.C.); chorazym@op.pl (M.C.); anna-mironczuk@wp.pl (A.M.); kochanowicz@vp.pl (J.K.); alakul@umb.edu.pl (A.K.); 2 Independent Statistical Consultant, 40-668 Katowice, Poland; fca.collin@gmail.com

**Keywords:** multiple sclerosis, disability progression, relapses, MRI

## Abstract

The aim of the study was to verify the association of clinical relapses and brain activity with disability progression in relapsing/remitting multiple sclerosis patients receiving disease-modifying treatments in Poland. Disability progression was defined as relapse-associated worsening (RAW), progression independent of relapse activity (PIRA), and progression independent of relapses and brain MRI Activity (PIRMA). Data from the Therapeutic Program Monitoring System were analyzed. Three panels of patients were identified: R0, no relapse during treatment, and R1 and R2 with the occurrence of relapse during the first and the second year of treatment, respectively. In the R0 panel, we detected 4.6% PIRA patients at 24 months (*p* < 0.001, 5.0% at 36 months, 5.6% at 48 months, 6.1% at 60 months). When restricting this panel to patients without brain MRI activity, we detected 3.0% PIRMA patients at 12 months, 4.5% at 24 months, and varying from 5.3% to 6.2% between 36 and 60 months of treatment, respectively. In the R1 panel, RAW was detected in 15.6% patients at 12 months and, in the absence of further relapses, 9.7% at 24 months and 6.8% at 36 months of treatment. The R2 group was associated with RAW significantly more frequently at 24 months compared to the R1 at 12 months (20.7%; *p* < 0.05), but without a statistical difference later on. In our work, we confirmed that disability progression was independent of relapses and brain MRI activity.

## 1. Introduction

Multiple sclerosis (MS) is the most common cause of disability in young adults, and affects 2.8 million patients worldwide [1]. The Expanded Disability Status Scale (EDSS) estimates disability progression in relapsing/remitting multiple sclerosis (RRMS) patients, but perhaps does not reveal all disability progression [2,3]. It is well-known that relapses contribute to neurological disability worsening in the short-term, but whether they contribute to long-term disability worsening is controversial [4,5]. In MS, the brain magnetic resonance imaging (MRI) activity to long-term disability is undetermined [5]. It was shown that, in patients with RRMS, the long-term disability progression occurred, which is independent of relapse and brain MRI activity [5].

The aim of the study was to determine relapses with disability progression in RRMS patients who were treated with disease-modifying therapies (DMTs) in Poland. We also assessed dependency between brain MRI activity and disability progression. We used the term disability worsening to describe an increase in disability in patients with relapse, while the term disability progression for patients in whom disability was independent of relapse and/or brain MRI activity [6,7]. All RRMS patients treated with DMTs in Poland were investigated based on data from the Therapeutic Program Monitoring System.

## 2. Materials and Methods

This observational, multicenter study with prospective data collection was performed in RRMS patients who were treated with DMTs reimbursed by the National Health Fund. The DMT drug programs are monitored and documented using the Therapeutic Program Monitoring System, a mandatory electronic health record system available on the National Health Fund website. Data from every patient treated in the drug programs were collected prospectively in the Therapeutic Program Monitoring System from 2014 to January 2018. The RRMS patients are included in the National Health Fund drug programs (first- or second-line) according to clearly described rules based on brain MRI activity, relapses, and disability progression [8,9]. Neurological examination including EDSS scoring was performed at baseline and then every 12 months. The EDSS score worsening was confirmed after 12 months by the treating neurologist with experience in MS care. Brain MRI was performed at baseline and then every 12 months in the local MS center, and reviewed by the radiologist and treating neurologist with experience in MS care according to current guidelines on the use of MRI in MS patients [10].

Brain MRI activity was defined as ≥ one new T2 lesion and/or ≥ one gadolinium enhancement (GD+) lesion with respect to previous brain MRI. A relapse was defined as new or recurrent neurologic symptoms not associated with fever or infection that lasted for at least 24 h. All relapses were recorded at the annual visit to monitor the effectiveness of treatment.

Demonstration panels were constituted to determine the correlations of clinical relapses and brain MRI activity with disability worsening/progression in the long-term compared to baseline. For the analysis, prescriptions were evaluated. In this study, the prescription designated more broadly the continuous sequence of annual observations since treatment start date for a given patient receiving a given drug. A prescription description includes patient characteristics (sex, age at DMT start), DMT start date, EDSS, drug name, treatment line, and end date, which might correspond to treatment interruption, last observation, or interruption in the follow-up.

Our study was approved by the Regional Medical Ethics Committee (the Medical University of Bialystok; Nr R-I-002/141/2018). Written consent to use the data for scientific research was granted by the president of the National Health Fund. Every patient undertaking treatment reimbursed by the National Health Fund consented to the collection of data in the Therapeutic Program Monitoring System. We received anonymized data without the ability to identify patients.

### Confirmed Disability Progression

The confirmed disability progression can be the result of relapse-associated worsening (RAW), progression independent of relapse activity (PIRA), or progression independent of relapses and brain MRI Activity (PIRMA). Disease worsening, defined as an increase of EDSS, was associated with relapse occurrence, whereas disability progression was associated with an increase of EDSS independent of relapse. Annually evaluated EDSS was compared to a fixed baseline reference EDSS score. Disability worsening/progression was acknowledged (binary response, yes/no) if the increase in EDSS was ≥1.5, ≥1, or ≥0.5 when baseline EDSS was <1, <6, or ≥6, respectively. It was not necessary to use a more sensitive methodology like a roving EDSS score to detect RAW and PIRA [3,11], because we assessed PIRA and RAW separately to exclude the relapse dynamic, which occurs during relapse and soon thereafter. A roving EDSS is independent of the baseline, and is more sensitive for increased detection analysis of PIRA in the group of MS patients with relapses.

The study focused on how relapses contributed to disability worsening over the long-term. Relapses were considered a treatment response after at least six months of treatment. The severity of relapse was described as low or high. A low relapse was defined as a 0.5-point increase in EDSS assessment and high as an increase of more than one point in the EDSS assessment. Based on the occurrence of relapses, three panels of evaluations were defined. The first panel grouped prescriptions without any relapses during treatment, and was designated as R0 (no relapses). Prescriptions were attributed to panel R1 in the case of a single relapse that occurred during the first year of treatment (6 to 15 months since prescription start). Similarly, prescriptions were attributed to panel R2 in the case of a single relapse that occurred during the second year of treatment. Prescription evaluations were included until treatment interruption, last observation, or interruption in the follow-up. The number of available observations declined with time.

Statistical analysis consisted of estimating the probability for disease worsening/progression (p). Logistic models were fitted against the time of observation (Did the prescription duration affect disease worsening?) and included the drug or treatment line (What was the effect of each drug on the disease course?) plus two-way interaction (Did the prescription duration effect depend on the prescribed drug?). The logit link function was used for linearization, approximated Chi-squared tested for observation time, drug, and interaction effect. The model relied on binomial distribution, or pseudo-binomial in the case of residual over-dispersion. Once the model was validated, Tukey’s procedure for post hoc multiple comparisons was used to test the differences in disease worsening probability between drugs and/or time point. Note that the logistic regression approach was preferred over survival analysis as to estimate the probability or frequency of disease disability worsening/progression regarding the time of evaluation and accounting for confounding factors, as well as possible remission with time.

The standard error included in the graphics was estimated as p×(1−p)/n, with p being the number of positives and *n* being the number of observed prescriptions. The analysis is presented hereafter step by step, investigating first the disease worsening/progression in the absence of relapse (R0), then comparing the evolution of the disease when a relapse was recorded during the first year (R1), and finally, evaluating the effect of the time of relapse on disease course by comparison with a single relapse occurring during the second year (R2). Progression independent of relapses and brain MRI activity was analyzed using the same methods, selecting patients with continuous follow-up (at least one visit per year) and without new MRI brain lesions since the last visit. The analysis was performed with software R (version 3.6.3, R Core Team, 2020) for reproducibility; the analysis (code and datasets) was bundled into an open R package accessible at https://github.com/FCACollin/rpack_pira (accessed on 14 February 2021).

## 3. Results

Three panels of continually observed prescriptions were identified: R0, no relapse during treatment, and R1 and R2, with relapse occurrence during the first year and the second year of treatment, respectively. Observational data were generally abundant at one year after the prescription start and rapidly decreased because of relapse selection criteria and interruption in the treatment or follow-up. Patient characteristics are presented in Table 1. The median EDSS at baseline and at the time of observation appears quite stable. However, a substantial fraction of the prescriptions was associated with a disease progression status. The following sections will be aimed at understanding the evolution of disease progression status in the absence of relapses (R0) or in comparison to panels with relapse occurrence during either the first year (R1) or the second year (R2) of treatment.

### 3.1. Progression Independent of Relapse Activity (PIRA)

**Figure 1:**
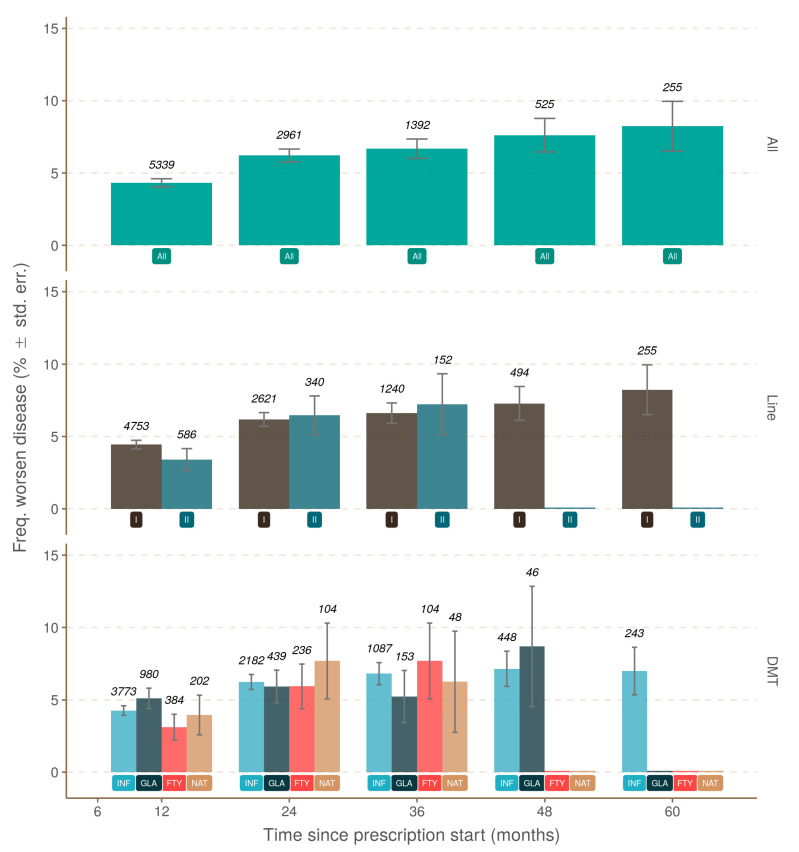
Comparison between observed frequencies of worsening diseases and month of observation when no relapse was ever recorded. The upper pane uses the complete set of observations (green; label at y = 1 is “All”), the middle pane uses first- and second-line treatment (label at y = 1; first-line, dark brown; second-line, dark blue), and the lower pane uses drug (label at y = 1; Fingolimod, red, FTY; Glatiramer Acetate, dark blue, GLA; Interferon, blue, INF; Natalizumab, light brown, NAT). Abundance is represented by transparency and the figure at the top of each bar. The error bar represents the standard error estimated as p × (1 − p)/*n*, with p being the number of positives and *n* the total number of observations. DMT: disease-modifying therapy.

The first panel was designated as R0, which described prescriptions without any relapses during treatment (Figure 1). A logistic model tested the actual disease progression over time, in which disease progression was confirmed (*p* < 0.001). The disease status of approximately 3.3% (standard error, SE = 0.26%) prescriptions was worse 12 months after starting the prescription compared to baseline (*p* < 0.001). The disease progressed significantly then from 12 to 24 months from prescription start (*p* < 0.001), with 4.6% of them being worse at 24 months (*p* < 0.001); the disease progressed further for 5.0% (36 months), 5.6% (48 months), and 6.1% (60 months) of prescriptions (Figure 1; Table 2). Although the increase beyond 24 months was not significant, this is likely linked to less abundant observations. No discriminating effect of the treatment line (*p* = 0.111) or prescribed drug (*p* = 0.215) on disease progression in the absence of relapse was found (Figure 1).

### 3.2. Relapse-Associated Worsening (RAW)

**Figure 2:**
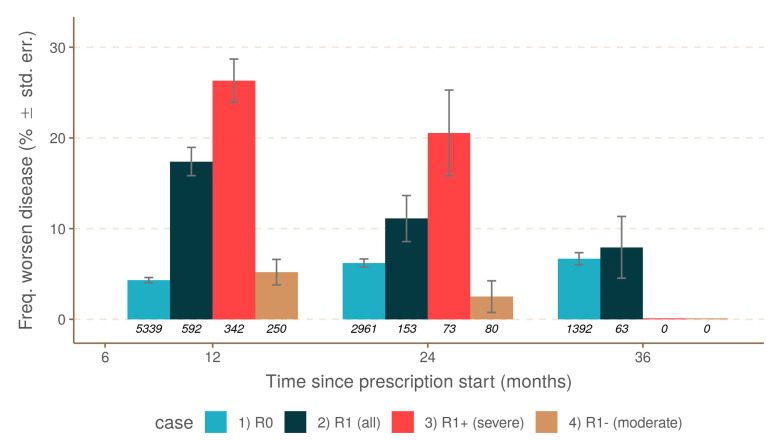
Comparison of disease worsening and one relapse during the first year of treatment (between 6 and 12 months). Colors are for: R0 = no relapse, R1 = relapse during the first year.

The prescription associated with relapse occurrence during only the first year of treatment and without other relapses during treatment (R1) was specifically studied in comparison to the R0 group. The number of observations corresponding to the criteria was much lower, and disease worsening was studied over three years after the prescription started. The logistic model showed a remission level after relapse occurrence depending on observation time (*p* < 0.001). Relapse occurrence in the first year of treatment was associated with 15.6% disease worsening at 12 months, significantly higher than in the group with the absence of relapse (*p* < 0.001) (Figure 2). In the absence of further relapses, recovery continued during the second year (9.7% at 24 months) but remained worse in comparison to the group without relapses R0 (*p* < 0.05). Finally, remission continued during the third year of treatment (6.8% at 36 months), possibly converging with the R0 scenario, i.e., a complete remission, although the reduced number of observations also reduced the confidence in this last estimation (Figure 2, Table 2). It is noteworthy that the discrimination of relapse occurrence during the first-year treatment based on relapse severity indicated large differences in disease evaluation at 12 months. Prescriptions from the R1 group with the occurrence of low-severity relapses were comparable to the R0 group. However, the number of observations was relatively low, and while the results tended to indicate that relapse severity mattered, this could not be tested by the present dataset.

**Figure 3:**
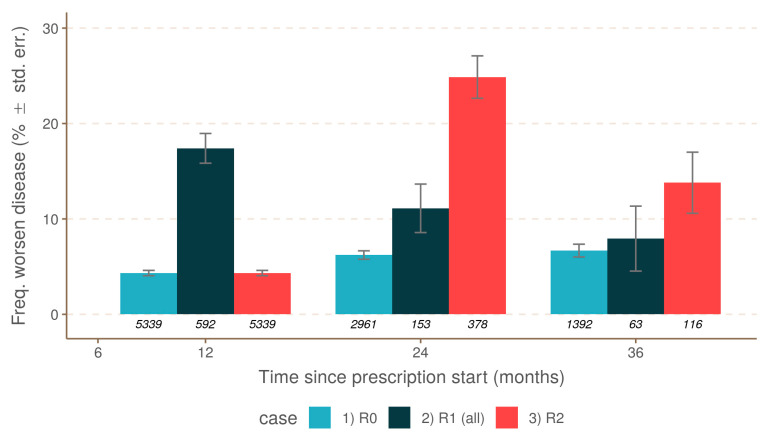
Comparison between disease worsening and three scenarios: R0 (no relapse), R1 (relapse during the first year), and R2 (relapse during the second year).

Disease worsening related to relapse depended on the year of its occurrence. To test it, a new logistic model was used to compare the R0 group to R1 and R2. Focusing on the R2 group, disease worsening was significantly more frequent (20.7%) when compared to R1 (15.6%, *p* < 0.05) (Figure 3). Nonetheless, remission one year after was finally associated with a significantly lower level of disease worsening (10.9%), without a statistical difference from the disease worsening in the R1 group (9.7%). This result was related to a much lower statistical power due to the lower number of observations corresponding to the identified scenario.

In our analysis, relapse occurrence was associated with clinically meaningful EDSS worsening at the annual examination, and gradually decreased due to remission in the subsequent annual examinations. Therefore, relapses were mostly associated with short-term, but not long-term, disability worsening due to prolonged recovery from relapses.

### 3.3. Progression Independent of Brain MRI Activity

**Figure 4:**
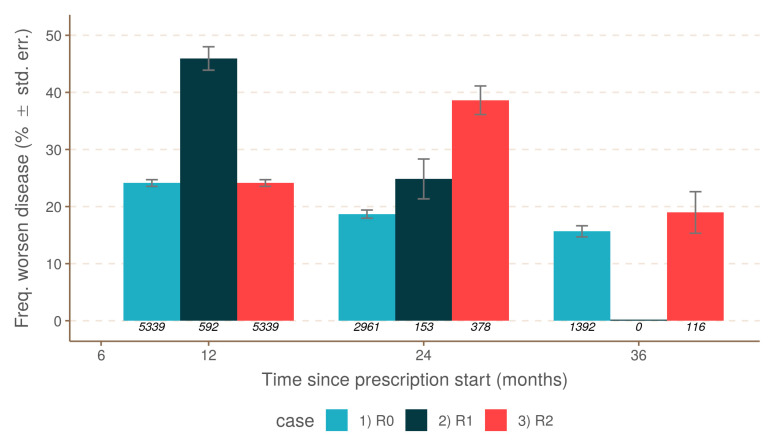
Comparison between MRI activity and the three scenarios: R0 (no relapse), R1 (relapse during the first year), and R2 (relapse during the second year).

**Figure 5:**
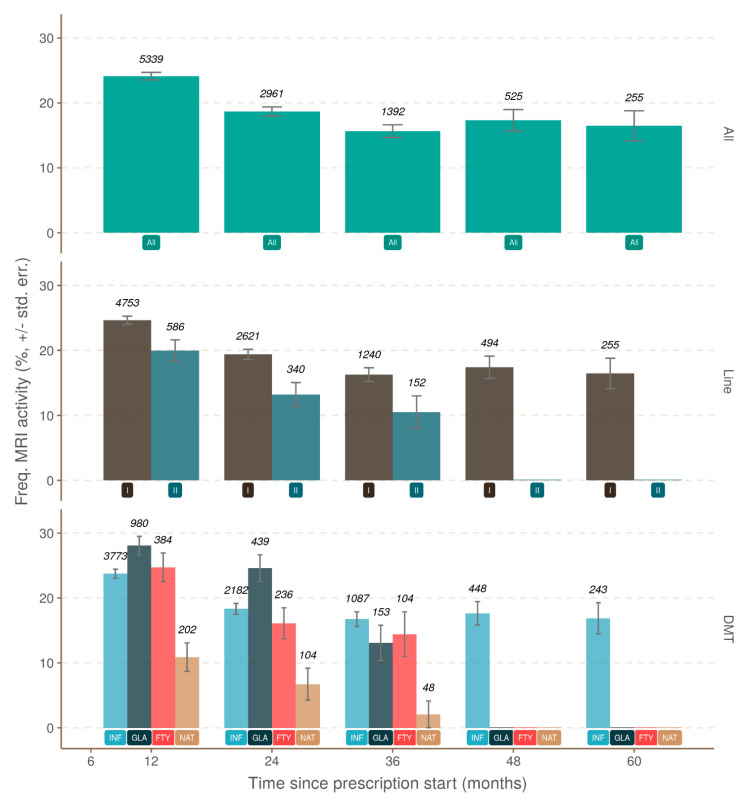
Comparison between the observed frequencies of MRI activity and month of observation when no relapse was ever recorded. The upper pane uses the complete set of observations (green; label at y = 1 is “All”), the middle pane uses first- and second-line treatment (label at y = 1; first-line, dark brown; second-line, dark blue), and the lower pane uses the drug (label at y = 1; Fingolimod, red, FTY; Glatiramer Acetate, dark blue, GLA; Interferon, blue, INF; Natalizumab, light brown, NAT). DMT: disease-modifying therapy.

As expected, brain MRI activity as defined by new brain lesions (T2 and/or with GD+) correlated strongly with the occurrence of relapses. Relapses during the first and second year (twelfth month and twenty-fourth month, respectively) of treatment were more frequently associated with brain MRI activity than disease progression (42% vs. 15.6%, 40% vs. 20.7%, respectively) (Figure 4). Brain MRI activity without relapse occurrence was also evaluated. In the absence of relapse, brain MRI activity decreased with the treatment duration. Brain MRI activity was more frequent at the beginning of treatment, and then decreased and stabilized after two years (Figure 5). There was a significant difference in brain MRI activity associated with the line and drug treatment. Lower brain MRI activity was under the second-line treatment, and natalizumab was the most effective drug to reduce brain MRI activity.

### 3.4. Progression Independent of Relapse and Brain MRI Activity (PIRMA)

**Figure 6:**
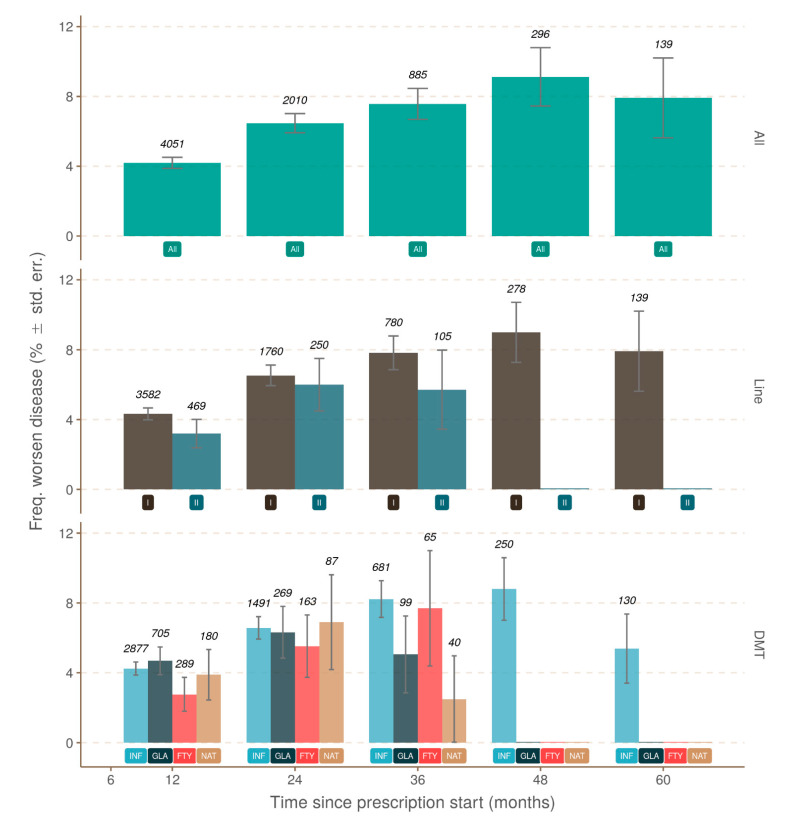
Patients with evidenced disease progression: the proportion of estimations derived from observation of patients never associated with any relapses or brain MRI activity between 12 and 60 months from the prescription start. The upper pane uses the complete set of observations (green; label at y = 1 is “All”), the middle pane uses first- and second-line treatment (label at y = 1; first-line, dark brown; second-line, dark blue), and the lower pane uses the drug (label at y = 1; Fingolimod, red, FTY; Glatiramer Acetate, dark blue, GLA; Interferon, blue, INF; Natalizumab, light brown, NAT). The error bar represents the standard error estimated asp × (1 − p)/*n*, with p being the number of positives and *n* the total number of observations. DMT: disease-modifying therapy.

A logistic model tested PIRMA over time. In the absence of relapses and brain MRI activity, the annual patient monitoring indicated disease progression. The proportion of patients with disease progression grew with time from the prescription start (*p* < 0.0001); however, it was not possible to detect a significant variation associated with either the drug choice (*p* = 0.625) or the treatment line (*p* = 0.315) (Figure 6). The proportion of patients with disease progression at 12 months was 3.0% (SE = 0.29%); it increased significantly to reach 4.5% (SE = 0.48%) at 24 months, and varied from 5.2% (SE = 1.60%) to 6.2% (SE = 1.24%) between 36 and 48 months from prescription start (Table 2).

## 4. Discussion

Our data revealed that PIRA and PIRMA occurred in RRMS patients living in Poland in a high-risk area for MS [12,13]. In MS patients, disease progression occurred early, even after a year of DMT treatment, and increased with the duration of treatment. Relapses during the first and second year were more frequently associated with brain MRI activity than with disease progression. No effect of the prescribed drug or treatment line on disease progression in the absence of relapse and/or brain MRI activity was evidenced.

In our work, RAW was related mostly to disability worsening over the short-term, but not with the risk of long-term disability. The incidence of RAW was confirmed in recent studies [7,11]. Some studies did not find the impact of relapses on long-term disability progression [5,14,15,16]. On the other hand, other studies showed that relapse frequency and recovery from relapses within the first few years of disease onset were related to long-term disability [5,17,18,19,20,21].

Whether the new lesions on brain MRI are related to long-term disability is controversial. Some studies have found that the number of lesions on initial brain MRI or detection of many new lesions due to relapses correlated with long-term disability [5,22,23], whereas other studies indicated that brain MRI activity correlates poorly with disability progression [5,24,25]. In our study, brain MRI activity was more frequent at the beginning of treatment, then decreased and stabilized after two years, and was not related to long-term disability progression.

In comparison, the distribution of disease progression revealed a relative independence from the duration of treatment. From the beginning, disability progression linearly increased in long-term follow-up. Our previous work showed that relapse and brain MRI activity as the first evidence of disease activity occurred 6–12 months after starting treatment with DMTs, and then progressively decreased. On the other hand, disease progression increased over the time of the MS course. During the long-term follow-up, a greater proportion of MS patients lost NEDA status (no evidence of disease activity) because of disease progression than because of relapses or brain MRI activity [8].

Our study has shown that, in RRMS patients, disability progression occurred independently of relapse, similar to recent studies [7,11]. Cree et al. showed that long-term disability progression is common in RRMS patients, was largely independent of relapse and focal lesion formation, and was associated with brain atrophy [5]. In this paper, Cree et al. showed that, in MS patients, brain volume loss occurs early, and is related to long-term disability, like in previous studies [5,26,27]. We do not know whether disability progression due to brain loss is dependent or independent of the focal lesions. The authors explained that either diffuse damage or, perhaps, focal lesions too small to be detected by brain MRI caused irreversible tissue loss. They also proposed the term “silent progression” to describe the insidious disability progression that occurs in many patients who fulfilled RRMS criteria [5]. These findings suggest that treatment inflammation is not sufficient for long-term outcomes of RRMS. In addition, there is a need to identify the other processes related to disability progression [28].

In most RRMS patients, the disease converted to a secondary progressive course [3,27]. Secondary progressive multiple sclerosis (SPMS) is characterized by an initial RRMS disease course, followed by progression of a variable rate with or without relapses [3,6,28,29]. SPMS is the strongest determinant of long-term disability progression [3,14,30,31]. Studies have suggested that disability progression occurs in early RRMS patients, and the loss of function is so gradual as to be unnoticed by either patients or physicians. These MS patients have low EDSS scores, and are, for the most part, fully functional, like in our study. Many neurologists did not recognize SPMS in patients with EDSS scores less than 3 [5]. A comprehensive analysis using a large MSBase cohort established an objective SPMS definition [32]. A certain reluctance remains in using this definition, which can result in the classification of patients after their EDSS scores increase as having RRMS [28]. A study using data from the Tysabri Observational Program of natalizumab-treated RRMS patients evaluated the metrics using fixed and roving EDSS baseline criteria for the identification of changes in disability unrelated to relapses. In this study, EDSS worsening was detected twice as often by the use of a roving EDSS reference [3]. A definition of SPMS due to disability progression unrelated to relapsed and/or brain MRI activity needs further studies that will include a large cohort of RRMS patients with a longer follow-up. Besides this, we should perform very careful observations of sustained disability progression not related to relapses and/or brain MRI activity in RRMS patients during treatment to prevent disability accumulation.

Studies have shown that using more effective drugs reduced conversion to SPMS compared with untreated patients [33]. In our work, there was a significant difference in brain MRI activity associated with drug treatment. Lower brain MRI activity was under the second-line treatment, and natalizumab was the most effective drug to reduce brain MRI activity. It was shown that treatment with a high-potency therapy reduced brain atrophy [5].

Our study has shown that long-term disability progression in RRMS is not only related to relapses and brain MRI activity. It is unclear whether MS patients with disability progression represent patients with RRMS or whether they have SPMS that has not yet been recognized [28]. The use of the EDSS scale combined with the measuring of upper limb function, walking speed, and cognitive function increases the number of patients with MS who have relapse-independent disability progression [5]. Additionally, measures of neurodegeneration, such as brain atrophy, could identify MS patients with slow disability progression [28]. It is crucial to recognize disability progression in RRMS patients to use more effective treatments that slow disability accumulation [34,35]. Additional therapy in combination with DMTs can be helpful. The modification of gut microbiota may serve as an additional therapeutic strategy in promoting treatment effectiveness and optimal response [36].

Our study has several limitations. Real-world studies have inherent limitations, such as confounding factors and the risk of a selection bias linked to the high number of patients lost to the follow-up due to missing patient visits. We assessed PIRA and RAW only based on EDSS. At the annual visit, EDSS, brain MRI activity, and relapses were registered. In our study, RRMS patients have been treated with many DMTs with different effectiveness. Despite these limitations, the results of this dataset are consistent with previously published studies. The consistency of our findings regarding short-term outcomes is a representative sample to long-term observations.

## 5. Conclusions

Our study has shown that long-term disability progression in patients with RRMS occurs even in the absence of clinical relapses and/or confirmed disease activity on MRI. It is crucial to recognize silent disability progression in RRMS patients to use more effective treatments that slow disability accumulation.

## Figures and Tables

**Table 1:** Patient characteristics.

Arm	M	*n*	F:M	Age	Age Symptoms	Symptoms	Relapse Number	EDSS Baseline	Worsening Number
R0	12	5339	2.32	36	30	3.61	0	1.5	231
R0	24	2961	2.24	37	30	3.78	0	1.5	184
R0	36	1392	2.2	36	30	3.38	0	1.5	93
R0	48	525	2.28	36	31	3.48	0	1.5	40
R0	60	255	2.04	36	31	3.16	0	1	21
R1	12	592	2.23	35	28	4.06	592	2	103
R1	24	153	2.48	35	28	4.37	0	2	17
R1	36	63	1.86	37	29	3.69	0	2	5
R1	48	21	2	32	28	2.57	0	1.5	0
R1	60	10	1.5	30	27	2.03	0	1.5	1
R1+	12	342	2.05	35	28	4	342	2	90
R1+	24	73	2.04	34	28	3.98	0	2	15
R1+	36	23	1.3	36	27	4.31	0	2	5
R1+	48	9	2	35	30	2.79	0	1.5	0
R1+	60	4	1	28	24	2.97	0	1.2	0
R1−	12	250	2.52	35	28	4.21	250	2	13
R1−	24	80	3	36	28	4.77	0	2.2	2
R1−	36	40	2.33	37	29.5	3.32	0	2	0
R1−	48	12	2	30	27	2.45	0	2	0
R1−	60	6	2	33	29	1.81	0	2	1
R2	12	5339	2.32	36	30	3.61	0	1.5	231
R2	24	378	2.26	37	30	3.46	378	2	94
R2	36	116	1.37	38	32	2.89	0	2	16
R2	48	31	2.1	38	32	2.16	0	1.5	5
R2	60	12	3	38	34	1.75	0	1.2	3
R0 + MRI0	12	4051	2.38	37	31	3.9	0	2	170
R0 + MRI0	24	2010	2.33	38	31	4.22	0	1.5	130
R0 + MRI0	36	885	2.16	38	31	3.78	0	1.5	67
R0 + MRI0	48	296	2.75	38	32	3.78	0	1.5	27
R0 + MRI0	60	139	2.56	38	32	3.65	0	1	11

Abbreviations: Arm = arm of the study; R0 = no relapse during treatment; R1, R2 = the occurrence of relapse during the first and the second year of treatment, respectively; MRI = magnetic resonance imaging; M = months; *n* = number; F:M = female to male ratio; Age Symptoms = age at the first symptoms; Symptoms = duration from the first symptoms to prescription start in years; EDSS = The Expanded Disability Status Scale; Worsening number = number of patients with disease worsening.

**Table 2:** Estimated adjusted means from the logistic regressions.

Arm	Time	Covariate	Prob	SE	Asymp.LCL	Asymp.UCL	Group
R1−	12	none	0.052	0.0140	0.030	0.087	a
R1−	24	none	0.025	0.0174	0.006	0.094	a
R1+	12	[EDSS = 2] [Sex = female]	0.226	0.0269	0.177	0.283	a
R1+	24	[EDSS = 2] [Sex = female]	0.165	0.0430	0.096	0.267	a
R1+	36	[EDSS = 2] [Sex = female]	0.173	0.0756	0.069	0.371	a
R0	12	[EDSS = 2] [Age = 30]	0.033	0.0026	0.027	0.037	a
R0	24	[EDSS = 2] [Age = 30]	0.046	0.0039	0.039	0.054	b
R0	36	[EDSS = 2] [Age = 30]	0.050	0.0055	0.039	0.061	b
R0	48	[EDSS = 2] [Age = 30]	0.056	0.0091	0.040	0.077	b
R0	60	[EDSS = 2] [Age = 30]	0.061	0.0133	0.039	0.092	ab
R0 + MRI0	12	[EDSS = 2] [Age = 30]	0.030	0.0029	0.024	0.036	a
R0 + MRI0	24	[EDSS = 2] [Age = 30]	0.045	0.0048	0.036	0.055	b
R0 + MRI0	36	[EDSS = 2] [Age = 30]	0.052	0.0162	0.040	0.068	b
R0 + MRI0	48	[EDSS = 2] [Age = 30]	0.062	0.0124	0.041	0.090	b
R0 + MRI0	60	[EDSS = 2] [Age = 30]	0.052	0.0160	0.028	0.094	ab
R1	12	[EDSS = 2] [Sex = female]	0.156	0.0177	0.123	0.193	a
R1	24	[EDSS = 2] [Sex = female]	0.097	0.0249	0.059	0.154	a
R1	36	[EDSS = 2] [Sex = female]	0.068	0.0301	0.027	0.156	a
R2	12	[EDSS = 2] [Age = 30]	0.031	0.0028	0.026	0.037	a
R2	24	[EDSS = 2] [Age = 30]	0.207	0.0212	0.168	0.251	b
R2	36	[EDSS = 2] [Age = 30]	0.109	0.0269	0.066	0.173	b
R2	48	[EDSS = 2] [Age = 30]	0.121	0.0528	0.049	0.267	b
R2	60	[EDSS = 2] [Age = 30]	0.178	0.0991	0.054	0.449	b

Abbreviations: Arm = arm of the study; R0 = no relapse during treatment; R1, R2 = the occurrence of relapse during the first and the second year of treatment, respectively; MRI = magnetic resonance imaging; EDSS = The Expanded Disability Status Scale; Time = time in months from the prescription start; Covariate = estimation adjusts for significant covariate at given levels. Prob = the estimated probability of disease worsening; SE = standard error of the estimated probability of disease worsening; Asymp. LCL = lower confidence level of the estimated probability of disease worsening; Asymp. UCL = upper confidence level of the estimated probability of disease worsening; Group = group comparison (within arm; a at 12 months, b at 24 months, and ab at 60 months; the difference is no longer significant (ab) due to the reduced number of subjects for which the estimation was made).

## Data Availability

The analysis (code and datasets) was bundled into an open R package accessible at https://github.com/FCACollin/rpack_pira (accessed on 14 February 2021).
